# The Macaque Cerebellar Flocculus Outputs a Forward Model of Eye Movement

**DOI:** 10.3389/fnint.2019.00012

**Published:** 2019-04-05

**Authors:** Gyutae Kim, Jean Laurens, Tatyana A. Yakusheva, Pablo M. Blazquez

**Affiliations:** ^1^Department of Otolaryngology, Washington University School of Medicine, St. Louis, MO, United States; ^2^Department of Neuroscience, Baylor College of Medicine, Houston, TX, United States

**Keywords:** forward models, cerebellum, motor control, oculomotor, Purkinje cell, cerebellar interneurons, mossy fibers

## Abstract

The central nervous system (CNS) achieves fine motor control by generating predictions of the consequences of the motor command, often called forward models of the movement. These predictions are used centrally to detect not-self generated sensations, to modify ongoing movements, and to induce motor learning. However, finding a neuronal correlate of forward models has proven difficult. In the oculomotor system, we can identify neuronal correlates of forward models vs. neuronal correlates of motor commands by examining neuronal responses during smooth pursuit at eccentric eye positions. During pursuit, torsional eye movement information is not present in the motor command, but it is generated by the mechanic of the orbit. Importantly, the directionality and approximate magnitude of torsional eye movement follow the half angle rule. We use this rule to investigate the role of the cerebellar flocculus complex (FL, flocculus and ventral paraflocculus) in the generation of forward models of the eye. We found that mossy fibers (input elements to the FL) did not change their response to pursuit with eccentricity. Thus, they do not carry torsional eye movement information. However, vertical Purkinje cells (PCs; output elements of the FL) showed a preference for counter-clockwise (CCW) eye velocity [corresponding to extorsion (outward rotation) of the ipsilateral eye]. We hypothesize that FL computes an estimate of torsional eye movement since torsion is present in PCs but not in mossy fibers. Overall, our results add to those of other laboratories in supporting the existence in the CNS of a predictive signal constructed from motor command information.

## Introduction

**Figure 1:**
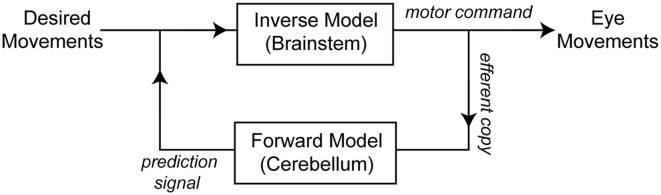
Schematic of the conceptual framework used for eye movement control (as in Green et al., 2007; Ghasia et al., 2008). An inverse model transforms desired movements into appropriate motor commands to move the eyes. A copy of the motor command is sent to a forward model that predicts the consequences of the motor command. The output of the forward model is sent back to the system where it is compared with the desired movement. A candidate structure for implementing the forward model of the eye movement is the flocculus complex (FL), and a candidate structure for the inverse model of the eye is the brainstem.

An important theoretical concept in motor control is that, for optimal motor performance, a control system must includes two internal models. One model converts the desired movement into forces (inverse model), while a second model works as a predictor that decodes the output of the inverse model (forces) into its consequences (forward or predictive model; Figure 1). These two internal models allow the control system to bypass the long delays associated with sensory feedback and adapt to variations in the environment (Wolpert et al., 1998). The forward model plays a pivotal role in maintaining accurate motor control because when its output is compared with the actual movement/sensory feedback, the result can be used to extract not-self generated sensation and to drive motor learning (Wolpert et al., 1998; Sawtell and Williams, 2008).

Neuroscientists have tried to apply this motor control theory to biological systems but finding the neuronal correlate of these internal models has proven difficult (Wolpert et al., 1995; Shadmehr et al., 2010). The existence of inverse models in biological systems is widely accepted because the brain must, somehow, convert desired movements into actual motor commands. However, the existence of biological correlates of forward models is still controversial. Accumulating evidence suggest that the brain uses forward models for motor control and point to a major role of the cerebellum in the construction of these forward models (Shadmehr and Mussa-Ivaldi, 1994; Wolpert and Kawato, 1998; Pasalar et al., 2006; Sawtell and Williams, 2008; Shadmehr et al., 2010; Brooks and Cullen, 2013). For example, cerebellar patients have impairments in perception during active movements suggesting a role of the cerebellum in the construction of sensory predictions of the consequences of motor command (Bhanpuri et al., 2012). Moreover, Purkinje cells (PCs) in cerebellar cortex lobules IV–VI of the non-human primate carry information related to both movement kinematics and error feedback, but not to motor command (Popa et al., 2012).

Eye movements are an ideal motor system to study motor control because of their simplicity when compared to other motor systems like arm movements. Eye movements are controlled by the action of three pairs of muscles and consist of rotations of the eye around three axes (horizontal, vertical and torsional). Interestingly, torsional eye movements during pursuit, saccades and ocular following are implemented by the mechanics of the orbit, not the motor command (Kono et al., 2002; Ghasia and Angelaki, 2005; Klier et al., 2011). Hence, torsion is present in the kinematics (actual movement) of the eye but not in the muscle dynamics (forces). This fact can be used as a powerful tool to search for neuronal correlates of forward and inverse models of the eye movement.

Ghasia et al. (2008) recorded the response of brainstem neurons during pursuit and found that putative flocculus-complex (FL) target neurons [eye head neurons (EH)], but not burst tonic neurons, carry torsional eye movement information during pursuit. They proposed that burst tonic neurons carry the output of the inverse model and FL target neurons carry the output of the forward model of the eye movement. Here, we test the hypothesis that the torsional eye movement signal detectable at the level of EH neurons originates in the FL. Furthermore, we test the hypothesis that this torsional signal is ultimately constructed in the FL. To test these hypotheses, we recorded the activity of PCs (output neurons) and mossy fibers (input elements) during similar pursuit tasks to those used by Ghasia et al. (2008). We present evidence suggesting that PCs carry torsional eye velocity information, but mossy fibers do not. These findings suggest that the torsional eye movement information found in FL target neurons in the vestibular nuclei arrives from the FL. Moreover, because torsional information is found at the output but not the input of the FL, we suggest that the FL plays an important role in the construction of forward models of the eye movement.

## Materials and Methods

### Animal Preparation and Experimental Setup

Two adult male rhesus macaques underwent two surgical operations to implant a scleral search coil, a titanium head-post, and a recording chamber. Recording chambers were implanted stereotaxically to record in the left FL, using zero tilt and pitch angles and with their centers aimed to 13 mm lateral and 1 mm posterior (Paxinos et al., 2000). Following a 3–4 weeks recovery period, we began training animals in oculomotor tasks using a standard water restriction protocol. All procedures conformed to the National Institutes of Health Guide for the Care and Use of Laboratory Animals and were approved by the Institutional Animal Care and Use Committee.

Animals were comfortably seated in a primate chair and on top of a rotating table (Kollmorgen, Radford, VA, USA) during our recording sessions. Animals were head fixed to the chair by their head posts to allow stable neuronal recordings. Our visual stimulus consisted of a red laser back-projected on a translucent screen located 50 cm in front of the animal. Vertical and horizontal laser positions were controlled using two mirror galvanometers that provided near linear displacement of the laser within the range used in this experiment: maximum deviation from linearity in +/− 20° range was 11.34% and 11.49% for horizontal and vertical directions, respectively. Horizontal and vertical eye positions were continuously measured using a three earth-fixed field coil system (CNC Engineering, Enfield, CT, USA). A reference coil was placed near the animals’ temporal bone and attached to the chair. The signal from the reference coil was subtracted from the eye coil signal to obtain the eye in head position. Neuronal data was filtered (bandpass 0.3–8 kHz) and amplified using an AC differential amplifier and headstage system (Model MDA-41 from BAK electronics, Umatilla, FL, USA). Eye, laser, and rotating table positions were recorded at a sampling rate of 0.5 KHz, and neuronal data at a sampling rate of 40 KHz using a power 1401 and spike2 software (Cambridge Electronic Design, Cambridge, UK).

### Behavioral Protocol

**Figure 2:**
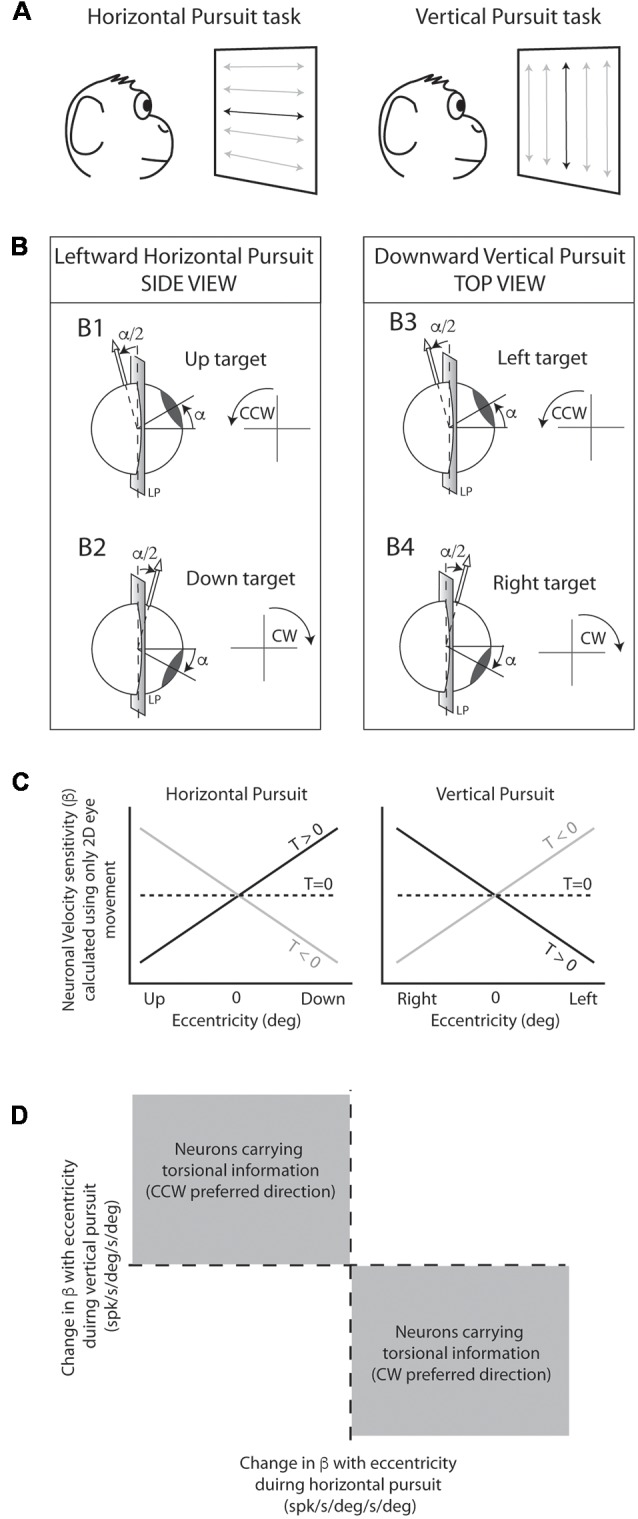
Experimental design and rationale. **(A)** Animals were seated in front of a projecting screen where a back-projected laser moved along specific paths. The main behavioral task consisted of sinusoidal horizontal (left) and vertical (right) pursuit, indicated by horizontal and vertical lines with arrow endings, respectively. Horizontal and vertical centered pursuit (indicated by the dark lines in the center of the projecting screen) consisted of pursuit eye movements around the center fixation point. Horizontal and vertical eccentric pursuit (indicated by the gray lines) consisted of horizontal and vertical pursuit eye movements that did not cross the center fixation point. **(B)** Schematic cartoon representing the torsional component of the eye movement during pursuit as predicted by the half angle rule. Leftward pursuit eye movements at up-gaze position generate counter-clockwise (CCW) torsional eye movements (B1) while leftward eye movements at down-gaze position generate clockwise (CW) eye movements (B2). Similarly, downward pursuit at left-gaze position generates CCW eye movements (B3) and downward pursuit at right-gaze position generates CW eye movements (B4). **(C)** Predicted changes in neuronal eye velocity sensitivity calculated using only 2D eye movements during the pursuit tasks shown in **(A)**. Neurons carrying 2D eye movement information would not change their eye velocity sensitivity with eccentricity (dashed line). Neurons carrying 3D eye movement information would change their eye velocity sensitivity with eccentricity as shown by the black and gray lines. Importantly, the change in eye velocity sensitivity with eccentricity (slope of the lines) provides information on the preferred torsional direction of the neuron (CW [*T* > 0] or CCW [*T* < 0]). **(D)** If we plot the slopes obtained in **(C)** (horizontal pursuit [left] vs. vertical pursuit [right]), neurons with CCW preferred directions would fall in the top left quadrant, while neurons with CW would fall in the bottom right quadrant.

All behavioral tasks were controlled by custom made software written in spike2 language. The eye coil was calibrated daily using 10–15° horizontal and vertical saccades. The main task used in this study consisted of sinusoidal smooth pursuit eye movements at different eccentricities similar to that used by Ghasia and Angelaki (2005). The laser was moved sinusoidally at 0.4 Hz and ±10° either in the horizontal or the vertical plane. This generated a laser peak velocity of about 25 deg/s. Horizontal pursuit tasks consisted of horizontal eye movements around the horizontal straight-ahead position at different vertical eccentricities (from +20 to −20°). Vertical pursuit tasks consisted of vertical eye movements around the vertical straight-ahead position at different horizontal eccentricities (from +20 to −20°; Figure 2A). We used the terms “centered horizontal pursuit” and “centered vertical pursuit” to refer to horizontal and vertical pursuit that pass through the center fixation point (straight-ahead position; black traces over the projecting screen in Figure 2A), and “eccentric horizontal” and “eccentric vertical” pursuit to refer to pursuit eye movements that do not pass through the straight-ahead position (gray traces over the projecting screen in Figure 2A). Animals were rewarded every 1–1.5 s with a small drop of water if they kept their eyes within a 3° distance from the moving target.

### Neuronal Recording

We recorded single units from FL, mostly ventral paraflocculus, using epoxy-coated tungsten microelectrodes (FHC Inc., Bowdoin, ME, USA, 8–10 MΩ impedance). The FL was identified by its characteristic eye-related activity. We identified the three layers of the cerebellar cortex using a standard procedure. The molecular layer was identified by the presence of complex spikes and the absence of simple spikes. The PC layer was identified by the presence of complex and simple spikes. When complex and simple spikes were recorded simultaneously, we further verified the identity of the recorded neuron (PC) and layer (PC layer) by detecting the complex spike-induced pause in simple spikes (>10 ms; Blazquez et al., 2003). The granular layer was identified by the absence of complex spikes and its characteristic saccade-related hashing activity. We commonly recorded two types of spikes in the granular layer: wide and narrow spikes. Wide spikes had similar width than simple spikes (>0.3 ms width) and typically showed a low firing rate. Narrow spikes (<0.25 ms width) typically showed clear saccade and eye position-related activity that matched the background hashing activity. The first type of spike is thought to be generated by granular layer interneurons, and the second by mossy fibers (Miles et al., 1980; Heine et al., 2010).

### Data Analysis

We followed the right-hand rule to define the positive and negative directions for horizontal, vertical and torsional eye movements (Ghasia and Angelaki, 2005; Klier et al., 2006): leftward, downward, and clockwise (CW) eye movements were considered positive, and rightward, upward, and counter-clockwise (CCW) eye movements were considered negative. The directionality of the movement (left/right; down/up; CW/CCW) was defined from the experimental subject point of view.

Spike sorting was performed off-line using analysis tools included in the Spike2 software (Cambridge Electronic Design, Cambridge, UK). Specifically, PC complex and simple spikes were sorted using a waveform template-match algorithm or a voltage threshold. Mossy fibers were first high-pass filtered (>400 Hz) and then sorted using a waveform template-match algorithm or a voltage threshold. Following this, data were exported to Matlab (MathWorks, Natick, MA, USA) for further analysis. In this study, we focused exclusively on the behavioral and neuronal responses to sinusoidal pursuit (0.4 Hz). The times corresponding to saccadic eye movements were detected using a 50 deg/s velocity threshold and removed from the behavioral and neuronal data. Following this, we computed the average behavioral (eye position and velocity) and neuronal response to several cycles of sinusoidal stimulation (at least five cycles). This average data was used for all subsequent analysis.

We fit the average response using a sinusoidal fitting function (0.4 Hz) in order to classify units as vertical or horizontal. Units were classified as horizontal if they showed a larger amplitude of modulation during centered horizontal pursuit than during centered vertical pursuit, and they were classified as vertical in the opposite case. Two horizontal mossy fibers, four horizontal PCs and two vertical PCs could not be recorded during centered pursuit. For these units, we characterized the neuronal directional preference and phase using the eccentric vertical and horizontal pursuit closest to the straight-ahead position. Neuronal response phase was defined with respect to peak eye velocity. We normalized the phases to the range of −90 to 90°, such that units that carry only eye position information would modulate their responses with a phase lag of 90 or −90°, while units that carry only eye velocity would modulate with a response phase of 0°.

Once a neuron was classified as horizontal or vertical unit, we extracted the neuronal sensitivities to eye position and eye velocity from the average neuronal responses using a standard linear fit procedure (Eq. 1, Model_PV_; Lisberger et al., 1994; Ghasia et al., 2008). Note that sinusoidal pursuit, the paradigm used in this study, is designed to extract the velocity information encoded in PC responses, which is the relevant signal for the question posed in this manuscript. Sinusoidal motion, however, cannot extract acceleration (as well as deceleration) and position signals independently because of cross-correlation effects (acceleration and position signals are 180° out of phase).

(1) $$FR=\beta *\dot{E}+\gamma *E+\delta +\varepsilon$$

where Ė and *E* correspond to the average eye velocity and position, respectively, *β* and *γ* to neuronal sensitivities to eye velocity and eye position, respectively, δ to the baseline (DC) firing rate, and ɛ the estimation error. These sensitivity values were calculated using horizontal eye movement information during horizontal pursuit and vertical eye movement information during vertical pursuit. Data where there was a change in eye movement in the orthogonal direction to the pursuit task direction (i.e., vertical eye movements during horizontal pursuit, or viceversa) of more than 1 deg/s within +20 and −20° eccentricity were excluded from further analysis. We selected this value arbitrarily, but such that it is much smaller (4–5 times) than the torsional eye velocity generated for the same change in viewing eccentricity (i.e., estimated torsional amplitude of +/−4.4 deg/s amplitude for a peak velocity of 25 deg/s, and +/− 20° eccentricity, see below half angle rule and Ghasia and Angelaki, 2005).

For each neuron, we estimated whether the eye velocity component contributed significantly to the neuronal response using a sequential *F*-test. First, we conducted a multiple linear regression using the *Model*_PV_ (eq. 1) and computed the sum of square of the regression, *SSR*_PV_, and the sum of squared errors, *SSE*_PV_, as follows:

$$SSR_{PV}=\sum_{i=1}^{n}{\left(Model_{PV}(i)-mean(Model_{PV})\right)}^{2}$$

$$SSE_{PV}=\sum_{i=1}^{n}{\left(FR(i)-Model_{PV}(i)\right)}^{2}$$

where *FR*, *Model*_PV_ and *n* are the neuronal responses, the reconstructed responses based on the regression, and the number of data points. Second, we conducted a multiple linear regression which contains only position component (i.e., we forced **β** = 0) and calculated the sum of square of the regression, *SSR*_P_:

$$SSR_{P}=\sum_{i=1}^{n}{\left(Model_{P}(i)-mean(Model_{P})\right)}^{2}$$

where *Model*_P_ is the regression model with only eye position component, and *n* is its total number. Lastly, a sequential test was performed by computing the following *F*-statistics:

$$F=\frac{(SSR_{PV}-SSR_{P})/m}{SSE_{PV}/\left(n-(k+1)\right)}$$

where *k* and *m* are the number of regression coefficients for *Model*_PV_ and *Model*_P_ (2 and 1, respectively). This value was compared with a Fisher distribution with *m* and *n* − (*k* + 1) degrees of freedom.

### Experimental Design and Rationale

Our experimental rationale is identical to that used by Ghasia and Angelaki (2005); Ghasia et al. (2008) and is based on two findings. First, horizontal pursuit above and below primary position, and vertical pursuit to the right and left of primary position generate torsional eye movements. The direction of the torsional eye velocity component can be predicted based on the eccentricity of the eye and the pursuit direction. Second, the torsional component of eye movements during pursuit is not represented in the motor command but implemented by the mechanics of the orbit (Demer, 2006; Klier et al., 2011). Thus, neurons that carry torsional eye velocity signal alone or in combination with horizontal and vertical eye velocity would modify their response depending on pursuit direction and eccentricity.

The rationale is explained graphically in Figure 2: the Listing law makes clear predictions about the magnitude and direction of torsional eye movements during pursuit. This is mathematically expressed by the half angle rule:

$$\dot{E}t=\dot{E}*tan(\alpha /2)$$

where Ė*t* is the torsional eye velocity, Ė is the eye velocity in 2D (horizontal and vertical) and α the eccentricity. During leftward pursuit, while the eyes are holding an upward gaze position the eyes move CCW (Figure 2B1), but the same leftward eye movement generates CW eye movements if the eyes are holding a gaze down eye position (Figure 2B2). Similarly, the half angle rule predicts CCW eye movements during downward pursuit while holding a leftward eye position, and CW eye movements during downward pursuit while holding a rightward eye position (Figures 2B3,4).

Next, let’s consider that the overall eye velocity sensitivity of a neuron can be represented by the following equation:

$$f(\dot{E})=\beta \nu *(\dot{E}\nu )+\beta h*(\dot{E}h)+\beta t*(\dot{E}t)$$

where *βv*, *βh*, and *βt* represent the neuronal sensitivities to vertical, horizontal and torsional eye velocity (spk/s/deg/s), respectively. Ė*v*, Ė*h* and Ė*t* the vertical, horizontal, and torsional eye velocities (deg/s), respectively. If we calculate the neuronal eye velocity sensitivity ignoring the torsional component of the equation [βt*(Ė*t*)], a neuron with no torsional information would have the same *f*(Ė) value during our pursuit tasks regardless of eccentricity (dotted lines in Figure 2C). However, a neuron with torsional eye velocity information (e.g., CW preferred direction [βt > 0]) would change *f*(Ė) during horizontal and vertical pursuit at different eccentricities (e.g., black lines in Figure 2C left and right panels).

Three important points are worth mentioning.

1. It could be difficult to determine whether a single neuron codes torsion because neuronal responses can be noisy, and the actual torsional eye movements during pursuit are small (Ghasia and Angelaki, 2005). However, at the population level, with a sufficiently large “*n*”, the presence of torsional information in the neuronal responses would be evident by having a significantly larger number of neurons falling in the gray areas shown in Figure 2D. We evaluate this statistically by performing a binomial test, which provides the likelihood of obtaining a particular number of successful draws (torsional coding neurons) given a total number of draws (total number of neurons) and assuming equal probability of getting successful and unsuccessful results in each draw (Figures 6, 10).
2. The sensitivity of our measurements is ultimately limited by the sample size (number of neurons) and the noise in the signal. We used computer simulations to determine the minimum torsional eye velocity sensitivity that can be detected using our analytical methods given our sample size and the noise in the signal (see Supplementary Figures S1, S2). The noise of the signal was calculated as the variation in firing rate within every single neuron for all tested eccentricities (−20, −10, 0, 10 and 20°); the noise was calculated separately for mossy fibers and PCs. Using the standard deviation of the noise, we created a normal distribution from which we randomly selected values representing gain change due to the noise of simulated neurons. The overall change in gain (eye velocity sensitivity) with eccentricity of a simulated neuron is equal to the gain change due to noise, plus the gain change due to the torsional eye velocity component. This idea can be represented mathematically as:

^Gain=n+βt*(E˙t)

where ^∧^Gain represents the gain change, *n* the gain change due to noise, *βt* the neuronal sensitivity to torsional eye velocity, and Ė*t* the torsional eye velocity. Torsional eye velocity is calculated directly from the half angle rule stated previously. A slope representing gain changes with eccentricity is calculated for horizontal and vertical pursuit (see Supplementary Figures S1A, S2A; same concept as in Figures 5, 9) and a binomial cumulative distribution function is used to look for whether the simulated population significantly represent torsion (located in second and fourth quadrant, Supplementary Figures S1B, S2B). We generate 100 iterations (simulated populations) with equal signal noise, sample size, and torsional eye velocity sensitivity, and obtain the percentage of iterations that significantly represent torsion (Supplementary Figures S1C, S2C). This process is repeated for different values of neuronal sensitivity to torsion generating a curve that represents, for a particular sample size and noise, how likely would it for our analytical methods to detect significant torsional signals (Supplementary Figures S1D, S2D). In the case of our mossy fiber population (*n* = 10), we could detect significantly torsional eye velocity sensitivities of 0.046 spk/s/deg/s in 95% of iterations. In the case of our horizontal and vertical PC populations (*n* = >18), we could detect significantly torsional eye velocity sensitivities of 0.035 spk/s/deg/s in 95% of iterations.

3. Although we do not record torsional eye movements, nor we calculate the true primary eye position (this would require knowledge of the actual torsion), because of the rules of ocular motility, we can be confident about how the torsional signal changes with eccentricity. That is, for horizontal pursuit, the more upward is the eccentricity, the more CCW is the torsion for leftward eye movements (viceversa for rightward eye movements). Similarly, for vertical pursuit, the more leftward is the eccentricity, the more CCW is the torsion for downward eye movements (viceversa for upward eye movements; Figure 2). Therefore, we can reliably tell the directionality of the changes in torsional eye velocity with pursuit direction and eccentricity. This information is a direct consequence of the Listing law and is sufficient to test our hypothesis.

**Figure 3:**
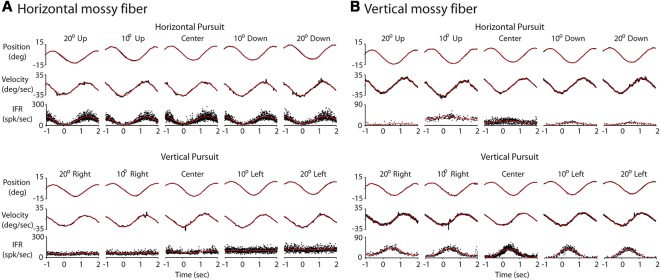
Response of two example mossy fibers during eccentric pursuit. **(A)** Response of a representative horizontal mossy fiber over several cycles of sinusoidal pursuit. The top three rows represent the average eye position (top, deg), average eye velocity (middle, deg/s) and mossy fiber response (bottom, spk/s) during horizontal pursuit at different vertical eccentricities. Neuronal response is shown by folding data (instantaneous firing rate) from multiple cycles into a single cycle. Each column represents one vertical eccentricity, in deg, from straight ahead-gaze (20 up, 10 up, 0, 10 down and 20 down). The bottom three rows show the same cell recorded during vertical pursuit at different horizontal eccentricities from straight ahead-gaze (20 right, 10 right, 0, 10 left and 20 left). The eye position and velocity data showed corresponds to either horizontal (top panels) or vertical (bottom panels) eye data. **(B)** The same as in **(A)** but for a representative example of a vertical mossy fiber. Red lines show the profile of the best fitting sinusoidal functions.

**Figure 4:**
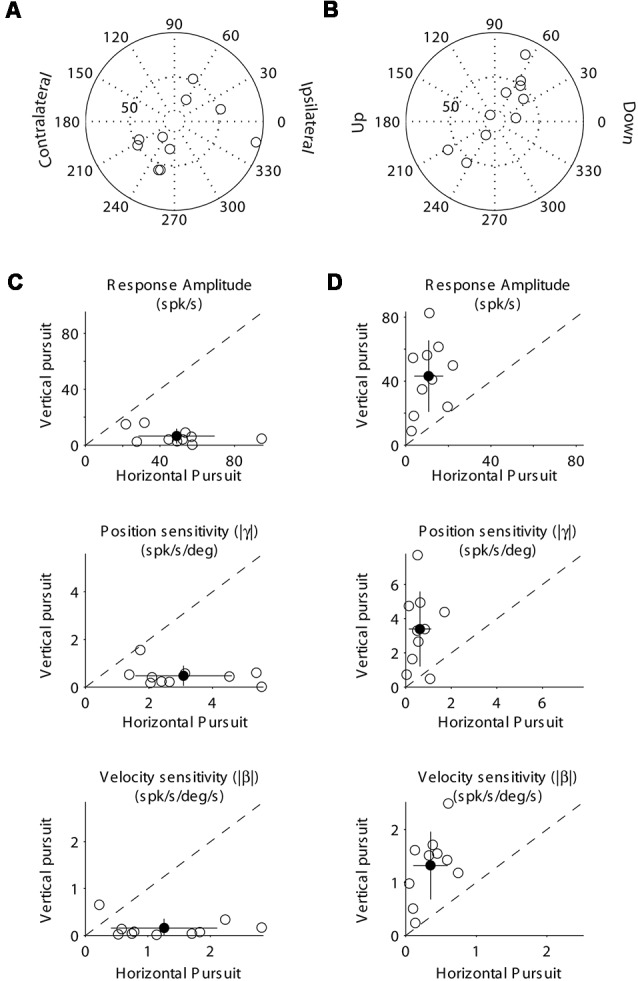
Population data showing the response modulation and neuronal sensitivity to eye movements of horizontal **(A,C)** and vertical **(B,D)** mossy fibers. **(A)** Polar plot showing the gain and phase of individual horizontal mossy fiber with respect to eye velocity during sinusoidal horizontal pursuit. Panel **(B)** same as **(A)** but for vertical mossy fibers during vertical sinusoidal pursuit. Panel **(C)** top, amplitude of neuronal modulation during horizontal vs. vertical sinusoidal pursuit. Middle, neuronal eye position sensitivity (absolute values) calculated during horizontal vs. vertical sinusoidal pursuit. Bottom, neuronal eye velocity sensitivity (absolute values) calculated during horizontal vs. vertical sinusoidal pursuit. Panel **(D)** same as **(C)** but for vertical mossy fibers. Filled symbols in **(C)** and **(D)** show the average values and the lines segments on top of them plus/minus one standard deviation from the mean.

**Figure 5:**
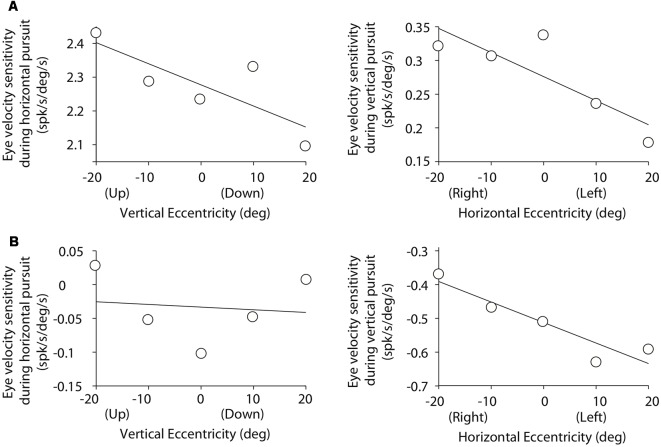
Cartesian plots showing the changes in mossy fiber eye velocity sensitivity with viewing eccentricity for the two example mossy fibers shown in Figure 3. **(A)** Data obtained from the example horizontal mossy fiber. Left plot shows the changes in eye velocity sensitivity during horizontal pursuit as we modified vertical viewing eccentricity (−20, −10, 0, 10, and 20°). Right plot shows changes in eye velocity sensitivity during vertical pursuit as we modified horizontal viewing eccentricity (−20, −10, 0, 10, and 20°). Panel **(B)** same for the example vertical mossy fiber.

**Figure 6:**
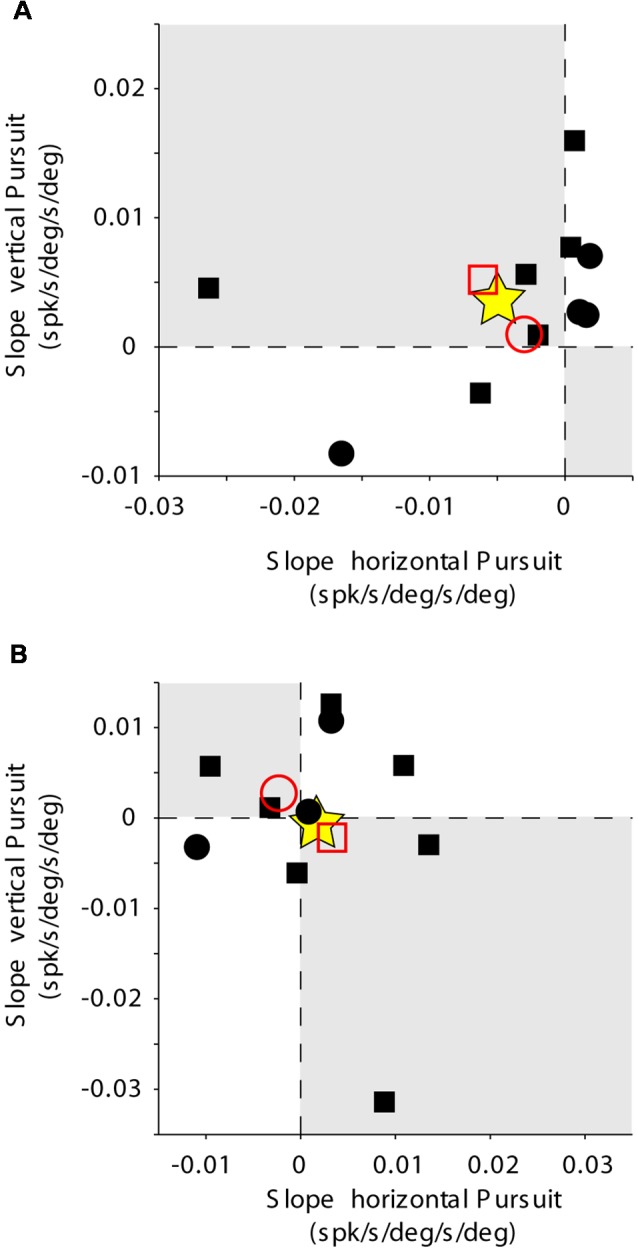
Mossy fiber population data. Rate of change of eye velocity sensitivity with eccentricity during horizontal pursuit (abscissa) vs. vertical pursuit (ordinate). The layout is identical to that shown for Figure 2D. **(A)** Data obtained for the population of horizontal mossy fibers. **(B)** Data obtained for the population of vertical mossy fibers. Each data point corresponds to a single mossy fiber. Empty red symbols represent the average value for each animal (animal 1 = circle, animal 2 = square) and stars the total average data.

**Figure 7:**
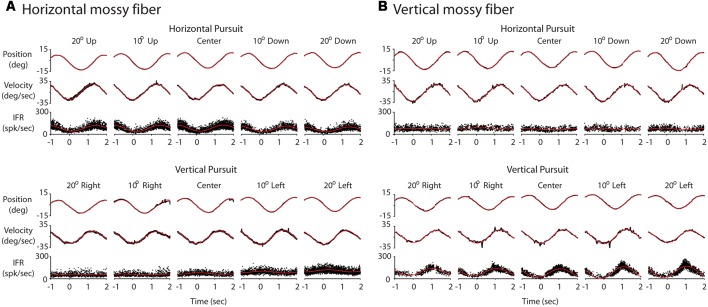
Response of two example Purkinje cells (PCs) during eccentric pursuit. The layout of the figure is identical to that of Figure 3. **(A)** Response of a representative horizontal PC averaged over several cycles of sinusoidal pursuit. **(B)** Response of a representative vertical PC averaged over several cycles of sinusoidal pursuit.

**Figure 8:**
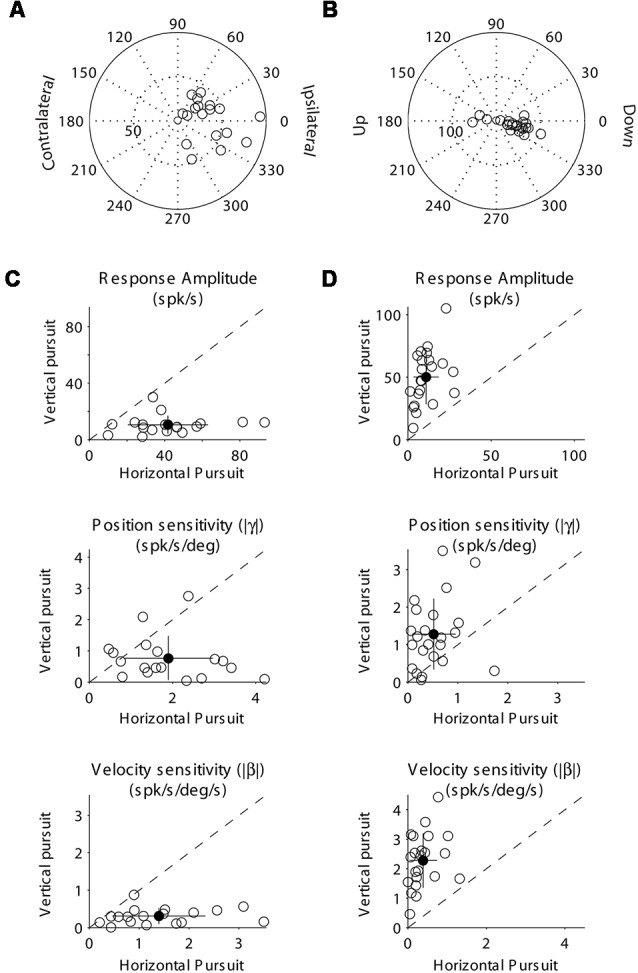
Population data showing the response modulation and neuronal sensitivity to eye movements of horizontal **(A,C)** and vertical **(B,C)** PCs. The layout is identical to that shown for Figure 4. **(A)** Polar plot showing the gain and phase of individual horizontal PCs with respect to eye velocity during sinusoidal horizontal pursuit. Panel **(B)** same as **(A)** but for vertical PCs during vertical sinusoidal pursuit. Panel **(C)** top, amplitude of neuronal modulation during horizontal vs. vertical sinusoidal pursuit. Middle, neuronal eye position sensitivity (absolute values) calculated during horizontal vs. vertical sinusoidal pursuit. Bottom, neuronal eye velocity sensitivity (absolute values) calculated during horizontal vs. vertical sinusoidal pursuit. Panel **(D)** same as **(C)** but for vertical PCs. Filled symbols in **(C)** and **(D)** show the average values, and the lines segments on top of them plus/minus one standard deviation from the mean.

**Figure 9:**
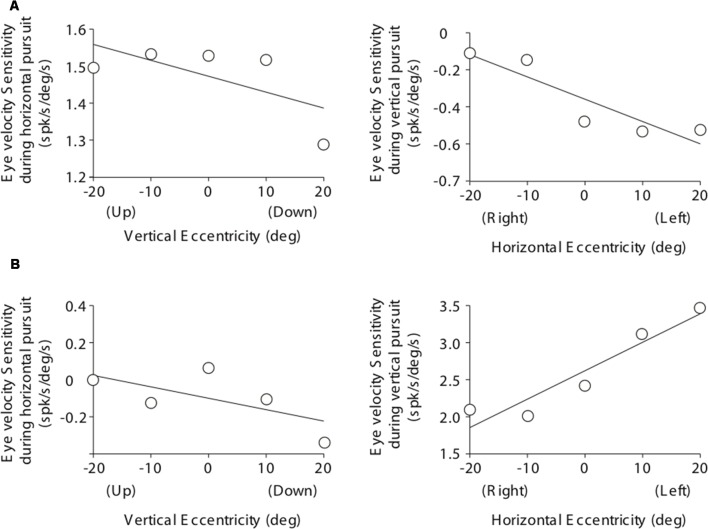
Cartesian plots showing the changes in PC eye velocity sensitivity with viewing eccentricity for the two example PCs shown in Figure 7. **(A)** Data obtained from the example horizontal PC. Left plot shows changes in eye velocity sensitivity during horizontal pursuit as we modified vertical viewing eccentricity (−20, −10, 0, 10, and 20°). Right plot shows changes in eye velocity sensitivity during vertical pursuit as we modified horizontal viewing eccentricity (−20, −10, 0, 10, and 20°). Panel **(B)** same as **(A)** but for the example vertical PC.

**Figure 10:**
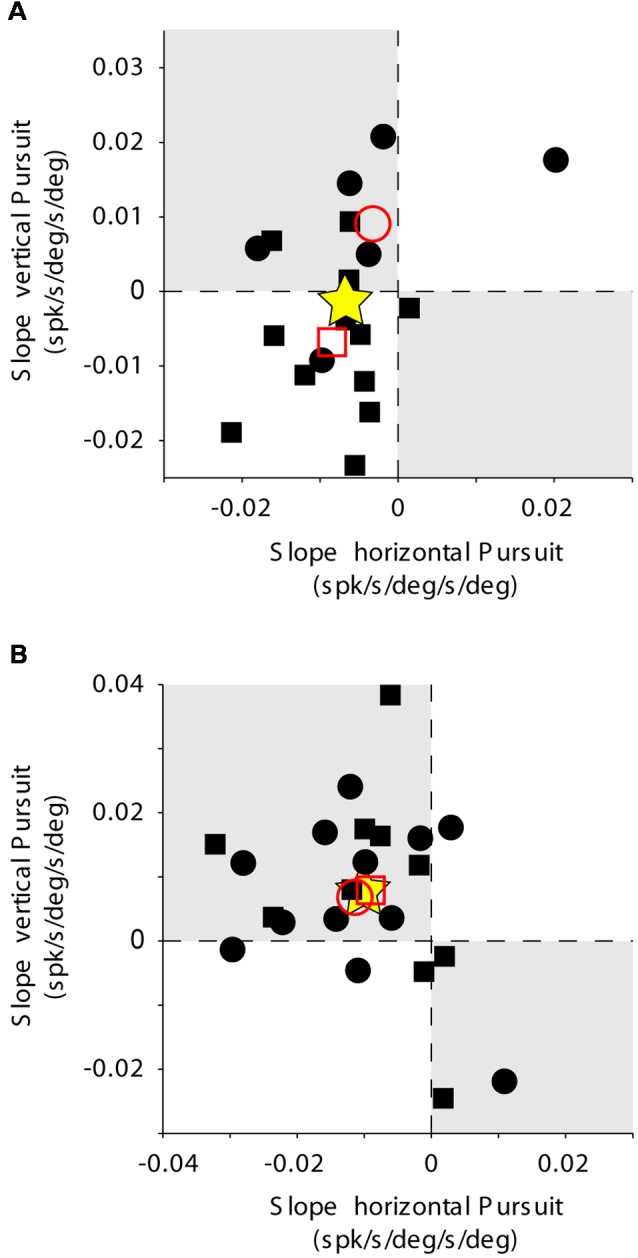
PC population data. The layout is identical to that shown for Figures 2D, 6. Rate of change of eye velocity sensitivity with eccentricity during horizontal pursuit (abscissa) vs. vertical pursuit (ordinate). **(A)** Data obtained for the population of horizontal PCs. **(B)** Data obtained for the population of vertical PCs. Each data point corresponds to a single PC. Empty red symbols represent the average value for each animal (animal 1 = circle, animal 2 = square) and stars the total average data.

## Results

We recorded the neuronal responses of 60 eye movement-related units (mossy fibers and PCs) in the FL of two macaque monkeys during horizontal and vertical smooth pursuit eye movements. Twenty units were classified as mossy fibers and 40 as PCs. Mossy fibers were identified based on their recording location (granular cell layer) and their characteristically narrow spike width (median of 0.25 ms for mossy fibers vs. 0.43 ms for PCs, respectively; *p* < 0.01, two-tailed *t*-test; Heine et al., 2010).

### General Mossy Fiber Responses During Pursuit

We recorded 10 horizontal and 10 vertical mossy fibers during the horizontal and vertical sinusoidal pursuit at different eccentricities. The mossy fiber shown in Figure 3A was classified as horizontal mossy fiber because it showed stronger modulation during horizontal centered sinusoidal pursuit (53.8 spk/s) than during vertical centered sinusoidal pursuit (9 spk/s). Its response phase during the horizontal centered sinusoidal pursuit was 15° (leftward preferred direction), indicating that this neuron carried eye velocity and eye position information. Indeed, the eye position and eye velocity sensitivity of this neuron during horizontal centered sinusoidal pursuit were 1.4 spk/s/deg and 2.2 spk/s/deg/s, respectively. The example mossy fiber shown in Figure 3B had an amplitude of modulation of 3.8 spk/s and 18.3 spk/s during the horizontal and vertical centered sinusoidal pursuit, respectively. Hence, it was classified as a vertical mossy fiber. Its modulation phase during the vertical centered sinusoidal pursuit was 57° (upward mossy fiber). Its eye position sensitivity (1.6 spk/s/deg/s) was more than three times larger than its eye velocity sensitivity (0.5 spk/s/deg/s). However, the eye velocity component played a significant role in shaping the neuronal response of this example mossy fiber (*p* < 0.01, partial *F* test).

At the population level, we found that horizontal mossy fibers could have either ipsilateral (*n* = 4) or contralateral (*n* = 6) directional preference with responses lagging their eye velocity (normalized values with respect to the preferred direction [−90 to 90°] of median 57.3°; mean 46.7°; STD 30.7°; Figure 4A). Similarly, vertical mossy fibers could have upward (*n* = 4) or downward (*n* = 6) directional preferences with responses lagging eye velocity (normalized values with respect to the preferred direction of median 55°; mean 38.2°; STD 37.2°; Figure 4B). Importantly, mossy fibers with a larger amplitude of modulation along a particular axis (e.g., horizontal) also have larger eye position and eye velocity sensitivity for movements along the same axes (Figures 4C,D). This further validates the method used to classify mossy fibers as horizontal and vertical units. The average sensitivity to eye position was 3.1 (STD 1.5) and 3.4 (STD 2.2) spk/s/deg for horizontal and vertical mossy fibers, respectively, which was more than twice the average sensitivity to horizontal and vertical eye velocity (1.25 [STD 0.9] and 1.3 [STD 0.6] spk/s/deg/s, respectively). This result agrees with previous work showing that mossy fibers carry both eye position and eye velocity information, but that their response is generally dominated by their eye position component (Miles et al., 1980). Nonetheless, the eye velocity component contributed significantly to the neuronal response of all recorded mossy fibers (*p* < 0.01, partial *F*-test).

### Mossy Fiber Responses During Pursuit at Different Viewing Eccentricities

Figure 5 illustrates the changes in eye velocity sensitivity with eccentricity for the example mossy fibers shown in Figure 3. The example horizontal mossy fiber (Figure 3A) had larger eye velocity sensitivities during the horizontal pursuit at upward eccentricities than at downward eccentricities (left panel of Figure 5A), and during the vertical pursuit at rightward eccentricities than at leftward eccentricities (right panel of Figure 5). Similar slope directions were found for the example vertical mossy fiber (Figure 5B). Following the rationale explained in our experimental methods (Figure 2), our example mossy fibers would not carry torsional eye movement information.

The changes in eye velocity sensitivity calculated for a single mossy fiber could be the result of the inherent noise in the neuronal response. To evaluate this possibility, we looked at the population data (Figure 6). Only about one-third of our mossy fiber units (35%, 7/20) showed changes in eye velocity sensitivity with viewing eccentricity that agree with the presence of a torsional component in their pursuit response. This number was not significantly different from chance (*p* = 0.13, binomial cumulative distribution function). Moreover, if we analyze separately horizontal (Figure 6A) and vertical mossy fibers (Figure 6B), we found that for both cases the numbers of torsional coding and non-torsional coding units were not significantly different from chance (*p* = 0.17 for horizontal mossy fibers and *p* = 0.37 for vertical mossy fibers, binomial cumulative distribution function). Based on our mossy fiber sample size (*n* = 10) and inherent noise of the neuronal response, our analytical method could detect torsional eye velocity sensitivities as small as 0.046 deg/s (see “Materials and Methods” section), which is more than one order of magnitude smaller than the eye velocity sensitivity of mossy fibers to horizontal and vertical eye velocity. Thus, our results suggest that eye movement-related mossy fibers in the FL do not carry significant torsional eye velocity information during pursuit.

### General Purkinje Cell Responses During Pursuit

We recorded 18 horizontal and 22 vertical PCs during horizontal and vertical sinusoidal pursuit at different eccentricities. Figure 7 shows the response of one representative horizontal (A) and one representative vertical (B) PC. Both example PCs showed responses dominated by eye velocity information, with amplitudes of modulation of 40.4 spk/s (A) and 66 spk/s (B), and phases of 28.9° (A) and −23.9° (B). Their eye position and eye velocity sensitivities were 1.7 spk/deg and 1.5 spk/deg/s, respectively, for the example horizontal PC (A), and −2.5 spk/s/deg and 2.4 spk/s/deg/s, respectively, for the example vertical PC (B).

At the population level, both horizontal and vertical PCs showed responses dominated by their eye velocity component as indicated by their response phases (median 18.1°, mean 9.6, STD 40° for horizontal PCs; and median −13.7°, mean −12, STD 10.9° for vertical PCs [normalized in −90 to 90°]; Figures 8A,B). Most PCs have ipsilateral or downward preferred direction (17 ipsilateral, 0 contralateral, 19 down and 3 up). Alike mossy fibers, the classification of PCs as horizontal or vertical was practically independent of the parameter used [amplitude of modulation (the parameter we used for classification), eye position sensitivity, or the eye velocity sensitivity]. Thus, horizontal PCs tend to have larger eye position and eye velocity sensitivity to horizontal eye movements than to vertical eye movements, while vertical PCs tend to have larger eye position and eye velocity sensitivity to vertical eye movements than to horizontal eye movements (Figures 8C,D).

The large influence of eye velocity information and preference for ipsi and downward directions in PCs contrasts with the large influence of eye position information and balance distribution of preferred directions in mossy fibers. This supports the hypothesis that the efferent copy information arriving at the FL undergoes spatial and temporal signal transformations within the cerebellar cortex (Miles and Braitman, 1980; Miles et al., 1980; Blazquez and Yakusheva, 2015).

### Purkinje Cell Responses During Pursuit at Different Viewing Eccentricities

The eye velocity sensitivity of horizontal and vertical PCs was differentially affected by viewing eccentricity. The example horizontal PC presented in Figure 7A showed, on average, lower values of eye velocity sensitivity during horizontal pursuit at downward eccentricities than during horizontal pursuit at upward eccentricities (slope: −0.0043; Figure 9A, left). Similarly, the eye velocity sensitivity is lower during vertical pursuit at leftward eccentricities than during vertical pursuit at rightward eccentricities (slope: −0.012; Figure 9A, right). At the population level, the rate of changes in eye velocity sensitivity with gaze eccentricity for horizontal PCs were in disagreement with the torsional coding hypothesis; eight neurons located in torsional coding areas and 10 neurons in not-torsional coding areas, which is not significantly different from chance (*p* = 0.4, binomial test; Figure 10A).

The example vertical PC shown in Figure 7B showed, on average, lower values of eye velocity sensitivity during horizontal pursuit at downward eccentricities (slope: −0.006), and during vertical pursuit at rightward eccentricities (slope: 0.04; Figure 9B). These slopes indicate that this PC could carry torsional eye velocity information (Figure 2). This finding was consistent at the population level. Vertical PCs were found in greater numbers in the torsional coding areas than in the non-torsional coding areas (81% [18/22] in torsional coding areas, which is significantly different from chance *p* < 0.0004, binomial cumulative distribution function). Moreover, most putative torsional cells had CCW preferred direction (15/18, Figure 10B).

Two additional findings support the presence of torsional coding information in the response of vertical PCs during pursuit. First, the average change in eye velocity sensitivity with eccentricity is similar in both animals (see empty red symbols in Figure 10B). Second, the average change in eye velocity sensitivity during horizontal pursuit along different vertical eccentricities was −0.01, and the average change in eye velocity sensitivity during vertical pursuit along different vertical eccentricities was 0.0072 (see star symbol in Figure 10), with a confidence interval for a 95% margin of error of 0.0052 and 0.0065, respectively. This confidently places the population results within the second quarter in Figure 10B, which corresponds to the CCW torsional direction. Based on our smallest PC sample size (*n* = 18) and inherent noise of the neuronal response, our analytical method could detect torsional eye velocity sensitivities as small as 0.035 deg/s (see “Materials and Methods” section), which is almost two order of magnitude smaller than the eye velocity sensitivity of PCs to horizontal and vertical eye velocity.

## Discussion

Current theories propose that in order to achieve fine motor control, the central nervous system (CNS) must construct a forward model of the movement (see Figure 1; Wolpert et al., 1998; Popa et al., 2012). Neuronal recordings and clinical studies have pointed to the cerebellar cortex as one candidate site where forward models are constructed (Ghasia et al., 2008; Bhanpuri et al., 2012). In this study, we evaluated this hypothesis by recording the activity of mossy fibers and PCs in the FL during sinusoidal smooth pursuit eye movements at different viewing eccentricities. We found that mossy fibers do not carry information related to torsional eye velocity, however PCs do. Our results agree with the hypothesis that mossy fibers carry the efferent copy of the motor command signal and PCs carry a processed signal that resembles the output of the forward model of the eye movement during pursuit (see Figure 1). Interestingly, only vertical PCs carry the torsional component of the eye movement. We hypothesize that the FL transforms oculomotor command signals into a prediction of the current state of the eye kinematics.

In these experiments, we did not record torsional eye movements, instead, we estimated the qualitative change in torsion using the half angle rule (see Figure 2; Demer, 2006). This is sufficient to evaluate whether the changes observed in PC and mossy fiber responses are indicative of them having 3D eye movement information. Moreover, although we did not calculate primary eye position (this would require knowledge of the actual 3D eye moment), the torsional component of eye movement does change in a predetermined qualitative way when comparing vertical pursuit with leftward and rightward eye position eccentricity, and horizontal pursuit with upward a downward eye position eccentricity (see Figure 2 and Kono et al., 2002; Klier et al., 2006). Because of all the above, the experimental approach of this study is a valid methodology to evaluate the presence of forward models of the eye movement in the cerebellar cortex.

### Mossy Fibers Do Not Carry Torsional Eye Movement Information During Pursuit

The majority of eye-related mossy fibers arrive at the FL from the prepositus hypoglossi nuclei (horizontal mossy fibers) and the paramedian track nuclei (vertical mossy fibers; Langer et al., 1985b; Büttner-Ennever and Horn, 1996; Escudero et al., 1996). Neurons in these nuclei carry eye position and eye velocity information (Escudero et al., 1996), and have dynamical response properties identical to those of motoneurons (Green et al., 2007). Hence, these mossy fibers carry an efferent copy signal to the FL. Our population of horizontal mossy fibers has an average response phase of about 47°, which is within the values reported for prepositus hypoglossi and medial vestibular neurons projecting to the FL (42°, Escudero et al., 1996; and 53°, Green et al., 2007; assuming that eye movement is perfectly out of phase with head during VOR). The directional preference of our eye-related mossy fibers was independent of the parameter used for their characterization (amplitude of response, eye velocity, or eye position sensitivity). Thus, indicating that they carry information related to a specific type of eye movement, like motoneurons or prepositus hypoglossi nuclei neurons do. Also, supporting that our population of mossy fibers represent the efferent copy pathway, we found a similar number of mossy fibers with ipsilateral preferred direction than mossy fibers with contralateral preferred direction, which is in perfect agreement with the known bilateral projection of the vestibular and prepositus hypoglossi nuclei to FL. In addition to the brainstem nuclei cited above, the pontine nuclei could also send efferent copy information to the FL (Ono et al., 2004).

Our results are in perfect agreement with the interpretation shown in Figure 1. Specifically, we found no evidence for a torsional eye velocity component in the response of eye-related mossy fibers. This was true even when we separated our population of mossy fibers between those with horizontal and vertical preferred directions. Our results add to the evidence from MRI and electrophysiological studies suggesting that the torsional component generated during pursuit and saccades eye movements is entirely generated by the mechanics of the orbit, not the motor command (Ghasia and Angelaki, 2005; Demer, 2006; Klier et al., 2011).

### Purkinje Cell Carry Torsional Eye Movement Information During Pursuit

To our knowledge, the response of PCs to torsional eye movements have not been investigated to date, however it has been reported that electrical stimulation of the FL generates extorsion of the ipsilateral eye (outward rotation of the eyes about an axis that coincides with the direction of gaze in primary position, Sato et al., 1991). It has also been reported that injection of muscimol generates intorsion of the ipsilateral eye (Chin et al., 2002). These results suggest that PCs play an important role in the control of torsional eye movements and are in perfect agreement with our findings. The majority of our putative torsion coding vertical PCs had CCW (16/18) preferred directions, which correspond to extorsion-preferred direction because they were recorded in the left FL. Anatomical data also support our results (Fukushima and Kaneko, 1995). Vertical PCs inhibit ipsilateral secondary vestibular neurons that receive inputs from ipsilateral anterior semicircular canal afferents and that are responsible for generating compensatory eye movements to ipsilateral head roll turns; therefore, for generating intorsion of the eye. Increased activity in vertical FL PCs would increase inhibition of their target neurons in the vestibular nucleus, therefore, generating extorsion. In the other hand, inactivation of the FL would remove tonic inhibition and would generate intorsion.

Our results also suggest that horizontal PCs do not carry a signal related to torsional eye movement. This result is also expected based on anatomy. Horizontal eye movements are controlled almost exclusively by the lateral and medial rectus muscles, which are eye muscles that do not participate in the active generation of torsional eye movements.

### How can the Cerebellar Cortex Compute Torsion?

Because torsional eye movements during pursuit are not neuronally driven, but implemented by the mechanics of the orbit alone (Demer, 2006; Klier et al., 2011), the brain must reverse engineer the mechanics of the orbit neuronally in order to generate an estimation of torsion. What possible mechanism can do this? One possibility is that the input/output gain of the cerebellum is modulated by a context-dependent signal corresponding to the position of the eyes in the orbit. The more eccentric the eyes are in the orbit, the larger the effect on the input/output gain. Interestingly, the cerebellar cortex has the necessary elements to support the above-mentioned reverse engineering of torsion during pursuit.

Our hypothesis is that torsional information would be generated by a context-dependent regulation of FL output *via* granular layer interneurons. We have shown that large interneurons in the FL granular layer with low and high CV2 values (likely, unipolar brush cells [UBCs] and Golgi cells, respectively) show primarily eye-position-related responses (Heine et al., 2010; Laurens et al., 2013). Others have shown that changes in the tonic inhibition of granule cells can modify the gain (input/output) of the granular layer (Mitchell and Silver, 2003). We argue that changes in the level of tonic inhibition of granule cells by Golgi cells in an eye position dependent matter could ultimately modulate PC gain in a matter similar to the half angle rule. In support, we have shown that blockage of GABA-A receptors in the FL results in PC gain increases (Blazquez and Yakusheva, 2015). An alternative mechanism involves UBCs. UBCs are abundant glutamatergic interneurons in the vestibulo-cerebellum (Ruigrok et al., 2011) that receive direct input from mossy fibers and synapse into neighboring granule cells (Mugnaini et al., 2011). Tonic excitation of granule cells by UBCs in an eye position dependent matter could change the gain of the output of the granular layer by mechanisms like firing rate potentiation (Nelson et al., 2003). Lastly, cerebellar motor learning, perhaps using torsional retinal slip signal as the teaching signal, could help tune the added gains to properly implement the half angle rule in the response of vertical PCs.

### Implications of our Results for Current Theories of Motor Control

It is still unclear whether internal models operate in the CNS as shown in Figure 1, or whether the CNS uses other strategies to control movements. However, accumulating evidence suggests that the CNS builds a forward or predictive signal and that this signal plays a fundamental role in fine motor control (Wolpert et al., 1998; Shadmehr et al., 2010). Our results support the hypothesis that the cerebellar cortex is one place where the CNS generates predictions of the actual state of the motor system (kinematics) based on motor commands.

The cerebellum does not control movement directly, but it plays a modulatory role of the motor output. In support, movement onset usually precedes PC responses (Hirata and Highstein, 2001; Sánchez-Campusano et al., 2007). Moreover, the relation between cerebellar output and motor behavior varies depending on the behavioral state and the behavioral task. For example, during classical conditioning, interpositus neurons do not reliably encode the kinematics of the eyelid through the course of learning. Instead, their response gain is variable, and their response phase reverses (Sánchez-Campusano et al., 2007, 2009). Similarly, FL PCs do not show the same unique relation to eye movements during pursuit, VOR and cancellation of the VOR (Lisberger and Fuchs, 1978; Blazquez et al., 2003). Hence, it is not surprising to find differences in the relation between PC discharge and motoneuron response during two pursuit conditions: one engaging torsional eye movements and one not engaging torsional eye movements (pursuit along primary eye position and pursuit at eccentric positions, respectively).

A role of the cerebellum in predicting stimulus kinematics has also been proposed, but strong evidence is still lacking. Thus, Kettner and collaborators show that FL PCs may carry a signal related to predicted changes in target trajectory (Suh et al., 2000; Kettner et al., 2002), and Miles and colleagues show that Crus I PC responses correlate with the motion of a tracking moving target (Miles et al., 2006; Cerminara et al., 2009). The interpretation of these previous studies could, however, be confounded by eye movements. We have recently used a task where the stimulus tracking phase is free from contamination of eye movement-related signal. We showed that FL PCs do not respond to the motion of relevant visual stimuli (Blazquez et al., 2017). Hence, it is possible that the cerebellum, at least the motor cerebellum, mainly builds forward models of our movements, while cortical areas or non-motor areas of the cerebellum form forward models of relevant environmental variables (Maus et al., 2010; Cheong et al., 2012; Atmaca et al., 2013; Schmahmann, 2019).

Our results cannot inform on whether PC responses represent the output of the forward model (Green et al., 2007) or a signal indicative of unexpected events; e.g., motion and sensory information not directly generated by the motor command (Sawtell and Williams, 2008; Brooks and Cullen, 2013). Indeed, it is possible that during torsional VOR, when torsional eye movements are generated actively, the predictive signal found in this study would be canceled by torsional efferent copy signal arriving through mossy fibers. Thus, resulting in no appreciable response of PCs to torsional eye movements. One possibility is that the final forward model is formed at the level of FL target neurons (FTNs) in the brainstem by averaging their PC drive (Langer et al., 1985a). In fact, torsional information seems less scattered at the level of individual FTNs than that we found in the FL PCs (Ghasia et al., 2008). But, regardless of whether PCs carry the final output of the forward model or are one step upstream to it, our data strongly suggest that at least part of the computations necessary to construct the forward model of the eye is carried out by the FL.

One important concept in cerebellar physiology is that the cerebellum can function as an adaptable filter that generates forward models (predictions of the consequence of motor command) that will be used for rapid control of motor behavior (Miall et al., 1993). Forward models could play a role in noise cancellation as well as a role in detecting unexpected events (Porrill et al., 2013). But, because the mechanical properties of the motor system change over time due to growth, injury and disease, an ideal forward model must be adaptable and follow specific learning rules. Thus, the circuit implementing the model can learn to generate a new prediction guided by a teaching or error signal (Porrill and Dean, 2007). In the case of the cerebellum, this error and teaching signal corresponds to the climbing fibbers. The construction of a forward model of torsional eye movements must be thus learned and be adaptable. Our experiments were not designed to test this adaptability, however evidence of it can be found in 2D pursuit eye movements (Medina and Lisberger, 2008). The fact that we found torsional signals in vertical PCs, but not in horizontal PCs, is in agreement with current models of cerebellar cortex function that propose that the cerebellar cortex is organized in microzones that perform separate computations (Porrill et al., 2013).

According to Marr and Albus’ theory of cerebellar function, the cerebellar cortex is an ideal structure to generate context-specific computations (Marr, 1969; Albus, 1971). They proposed that the input layer of the cerebellar cortex samples the state of the motor system at any given time and generates a pattern of activity that contains contextual information. We believe that this general principle of Marr and Albus’ theory of cerebellar function is still valid today and can be readily applied to explain how the cerebellar cortex reverse engineer torsional eye movement information from 2D efferent copy information. According to our view, granular layer interneurons like Golgi cells would provide context-specific information that can modulate granule cell output, hence, PC responses (Heine et al., 2010; D’Angelo et al., 2013). This process, directed by cerebellar plasticity, could generate a signal that implements the half angle rule neuronally.

## Ethics Statement

All procedures conformed to the National Institutes of Health Guide for the Care and Use of Laboratory Animals and were approved by the Washington University Institutional Animal Care and Use Committee.

## Author Contributions

PB designed the experiments. GK, JL and PB carried out the data acquisition. GK and PB carried out the data analysis. PB, TY and JL carried out the manuscript preparation.

## Conflict of Interest Statement

The authors declare that the research was conducted in the absence of any commercial or financial relationships that could be construed as a potential conflict of interest.

## Supplementary Material

FIGURE S1 Simulation results for mossy fibers with a sample size of 10. **(A)** Example of a simulated neuron during horizontal (top) and vertical (bottom) pursuit, plotting difference in gain over the different eccentricities when torsional eye velocity sensitivity is 0.04 spk/s/deg/s. **(B)** Example of one of the populations simulated using torsional eye velocity sensitivity is 0.04 spk/s/deg/s. **(C)** Percentage of iterations (populations of 10 neurons) over 100 iterations that show significant torsional coding. **(D)** Changes in the percentage of iterations that show significant coding with changes in the torsional eye velocity sensitivity. Note that the larger the torsional eye velocity sensitivity, the most likely we could detect the torsional signal in the population.

FIGURE S2 Same as in Supplementary Figure S1 but for Purkinje cells with a sample size of 18.
