# Supplementary figures and images for: The Macaque Cerebellar Flocculus Outputs a Forward Model of Eye Movement
Source: Front Integr Neurosci. 2019 Apr 5;13:12. doi: 10.3389/fnint.2019.00012 (PMC6460257; doi:10.3389/fnint.2019.00012)

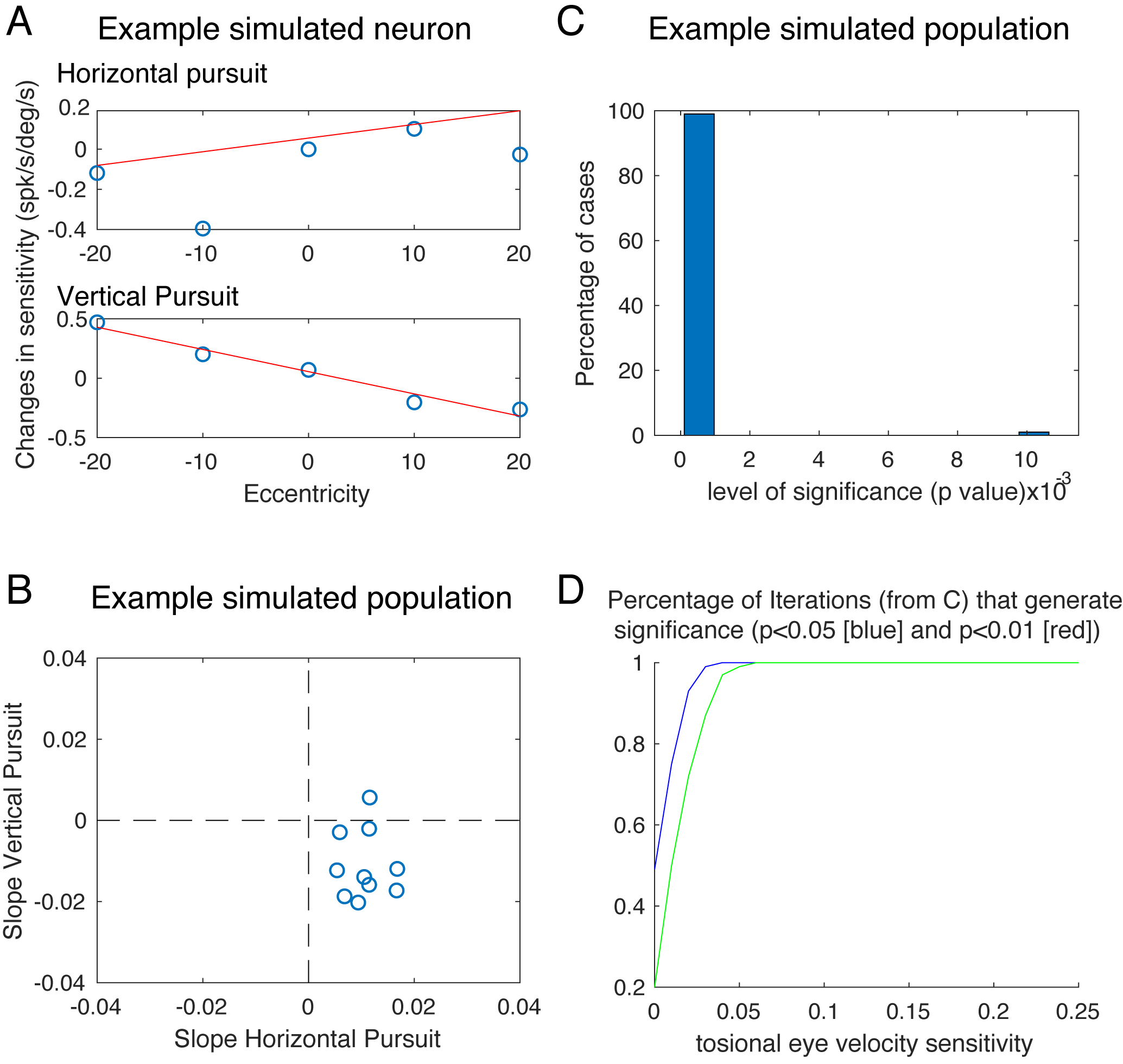

Supplement: FIGURE S1 — Simulation results for mossy fibers with a sample size of 10. (A) Example of a simulated neuron during horizontal (top) and vertical (bottom) pursuit, plotting difference in gain over the different eccentricities when torsional eye velocity sensitivity is 0.04 spk/s/deg/s. (B) Example of one of the populations simulated using torsional eye velocity sensitivity is 0.04 spk/s/deg/s. (C) Percentage of iterations (populations of 10 neurons) over 100 iterations that show significant torsional coding. (D) Changes in the percentage of iterations that show significant coding with changes in the torsional eye velocity sensitivity. Note that the larger the torsional eye velocity sensitivity, the most likely we could detect the torsional signal in the population. [file Image_1.TIF]

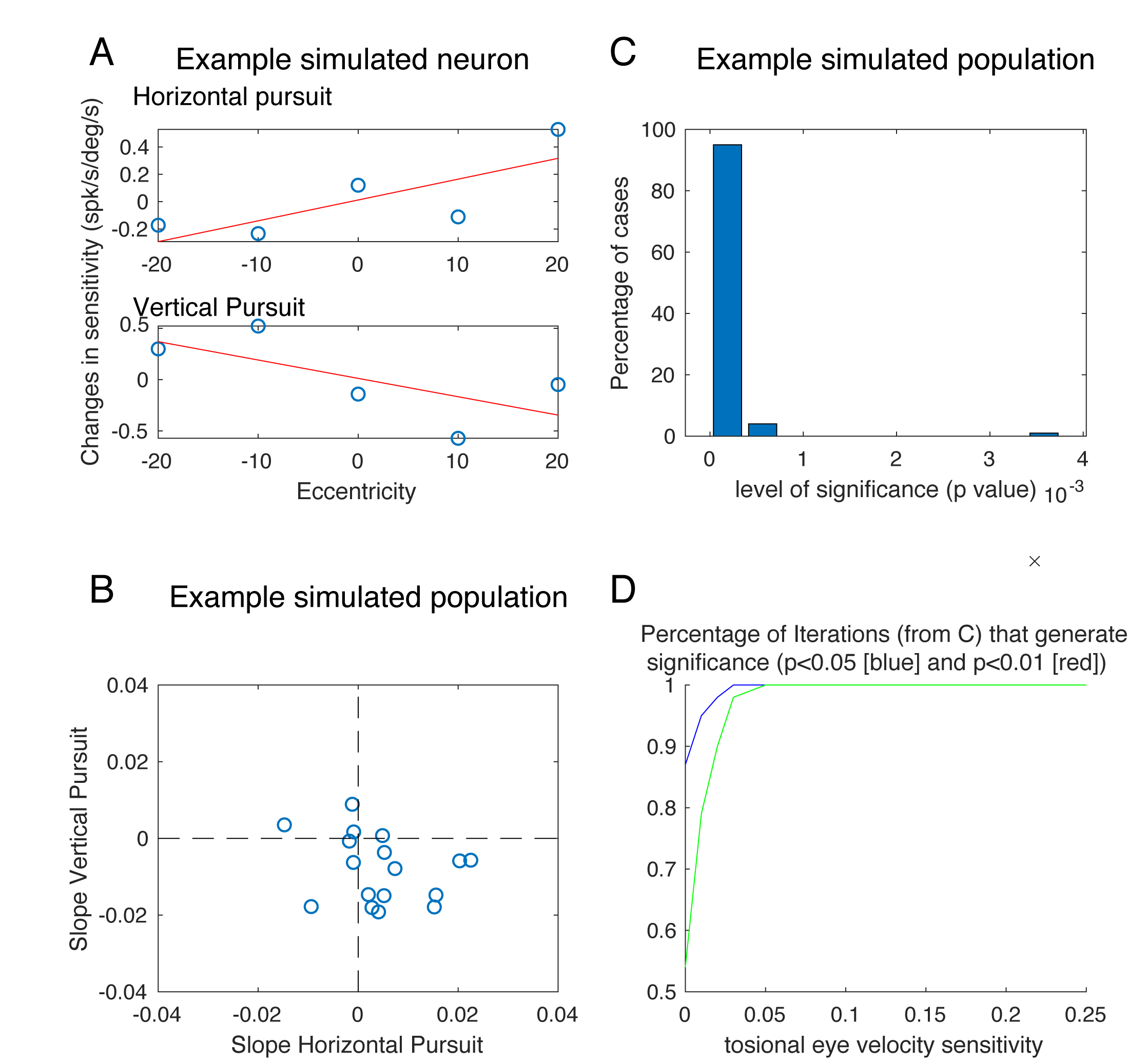

Supplement: FIGURE S2 — Same as in Supplementary Figure S1 but for Purkinje cells with a sample size of 18. [file Image_2.TIF]
